# New insights into disorders of gonad development using whole genome analysis

**DOI:** 10.1186/1687-9856-2013-S1-O2

**Published:** 2013-10-03

**Authors:** Andrew Sinclair

**Affiliations:** 1 Murdoch Children’s Research Institute and Dept. of Paediatrics, University of Melbourne, Australia; 2 Royal Children’s Hospital, Melbourne, Australia

## 

One of the most fundamental influences on our lives is our sex. Whether we are born as male or female has an enormous impact on our behaviour, reproductive options and disease susceptibility. Consequently, if the sex of a baby is not clear it can create many intractable issues, particularly in terms of medical management.

Disorders of Sex Development (DSDs) are congenital conditions in which development of chromosomal, gonadal or anatomical sex is atypical. The cause of these problems is most often a breakdown of the complex network of gene regulation responsible for proper development of testes or ovaries in the embryo. In the majority of DSD patients the etiology is unknown and they cannot be given an accurate diagnosis. We aim to identify the underlying changes in gonad genes of these DSD patients, provide a diagnosis and gain insights into gonad development.

We used microarrays to detect changes in copy number in DSD patients. This approach identified potential novel testis specific regulatory regions in SOX9, a known testis gene. We also showed that deletions and duplications affecting the regulatory sequences of SOX3 can cause it to be ectopically expressed in the developing gonad, allowing it to drive testis development in the absence of SRY in patients with 46,XX testicular DSD. In addition, we have identified mutations in the novel gene, MAP3K1 in patients with 46,XY DSD; implicating a new signal transduction pathway in testis development.

We’ve used Massively Parallel Sequencing (MPS) in three different ways. Firstly, we developed a rapid targeted MPS approach allowing in depth analysis of up to 200 gonad genes per DSD patient. Secondly, we used whole exome capture and MPS on several DSD families, trios and single cases. Finally, we employed whole genome MPS on trios with DSD.

This work demonstrates the tremendous power of whole genome approaches, especially when combing MPS data with linkage analysis of a large family. Whole genome analysis provides a rapid approach to identification of the disease-causing mutations and molecular diagnosis in patients with DSD as well as providing insights into gonad development and sex differentiation.

